# The mutagenic effect of tobacco smoke on male fertility

**DOI:** 10.1007/s11356-021-16331-x

**Published:** 2021-09-18

**Authors:** Temidayo S. Omolaoye, Omar El Shahawy, Bongekile T. Skosana, Thomas Boillat, Tom Loney, Stefan S du Plessis

**Affiliations:** 1 College of Medicine, Mohammed Bin Rashid University of Medicine and Health Sciences, Dubai, United Arab Emirates; 2 Division of Medical Physiology, Faculty of Medicine and Health Sciences, Stellenbosch University, Tygerberg, South Africa; 3 Department of Population Health, New York University Grossman School of Medicine, New York City, NY USA

**Keywords:** Tobacco smoke, Genetic aberrations, Mutagen, Aneugens, Spermatozoa, Male infertility

## Abstract

Despite the association between tobacco use and the harmful effects on general health as well as male fertility parameters, smoking remains globally prevalent. The main content of tobacco smoke is nicotine and its metabolite cotinine. These compounds can pass the blood-testis barrier, which subsequently causes harm of diverse degree to the germ cells. Although controversial, smoking has been shown to cause not only a decrease in sperm motility, sperm concentration, and an increase in abnormal sperm morphology, but also genetic and epigenetic aberrations in spermatozoa. Both animal and human studies have highlighted the occurrence of sperm DNA-strand breaks (fragmentation), genome instability, genetic mutations, and the presence of aneuploids in the germline of animals and men exposed to tobacco smoke. The question to be asked at this point is, if smoking has the potential to cause all these genetic aberrations, what is the extent of damage? Hence, this review aimed to provide evidence that smoking has a mutagenic effect on sperm and how this subsequently affects male fertility. Additionally, the role of tobacco smoke as an aneugen will be explored. We furthermore aim to incorporate the epidemiological aspects of the aforementioned and provide a holistic approach to the topic.

## Introduction

Globally, the prevalence of tobacco smoking remains high, with an estimated 19% of the adult population using tobacco products: 33% among males and 6% among females (World Health Organization 2019). These estimates are concerning as tobacco use has consistently been associated with numerous chronic diseases and adverse health outcomes, including possible mutagenic effects (World Health Organization 2020). There is a strong body of evidence suggesting a causal association between tobacco use and both cardiovascular- and cancer-related morbidity and mortality. A recent meta-analysis of 141 cohort studies reported that even low tobacco use (one cigarette per day) increased the risk of developing coronary heart disease by between 48 and 74% and smokers consuming 20 cigarettes per day were twice as likely to develop coronary heart disease (Hackshaw et al. 2018). Similarly, recent meta-analyses have reported a dose-response relationship between cigarette consumption and various types of cancer including bladder (Cumberbatch et al. 2016), kidney (Santucci et al. 2019), lung (O’Keeffe et al. 2018), ovarian (Santucci et al. 2019), pancreatic (Lugo et al. 2018), and prostate cancer (Islami et al. 2014). Cardiovascular and neoplastic health risks persist across other types of tobacco use including waterpipes (Montazeri et al. 2017) and smokeless tobacco (Sinha et al. 2018), and also exposure to environmental tobacco smoke (Zhang et al. 2020).

Not only has the association between tobacco smoke and cardiovascular- and cancer-related morbidity been shown; findings have also highlighted the adverse impact of tobacco smoke on male fertility (Sharma et al. 2016; Pizzol et al. 2021). Tobacco smoke contains several toxic and mutagenic substances, including nicotine, a psychoactive substance. Nicotine and its metabolite, cotinine, can cross the blood-testis barrier, which subsequently causes harm of diverse degree to the germ cells. Tobacco smoke has been reported to cause not only a decrease in sperm motility (although controversial), sperm concentration, and an increase in abnormal sperm morphology, but also abnormal protein expression, and both genetic and epigenetic aberrations in spermatozoa (Pereira et al. 2014). Studies have further provided evidence that tobacco smoke can serve as a mutagen and an aneugen of germ cell/spermatozoa (Marchetti et al. 2011; Linschooten et al. 2013; Pereira et al. 2014).

Although there is conflicting evidence on the mechanism of action of tobacco smoke on male fertility (du Plessis et al. 2014), the reported adverse effects ranging from the germlines to mature spermatozoa and overall male fertility cannot be overlooked. As such, the primary aim of this review is to provide an update on the evidence from animal and human research on the possible biological pathways through which tobacco use and exposure might cause adverse changes in sperm parameters leading to male infertility. Additionally, this review will also provide evidence that smoking has a mutagenic and an aneugenic effect on sperm and how this subsequently affects male fertility.

### Search methods

A literature search was performed of all relevant information related to tobacco smoking, its effect on human sperm parameters, and its role as a mutagen. Electronic databases including Google Scholar, PubMed, and the National Centre for Biotechnology Information (NCBI) were made use of to carry out these searches. Keywords such as “tobacco/cigarette smoking,” “sperm parameters,” “sperm DNA fragmentation,” “mutagen,” and “aneugen” were utilized to perform the searches. Literature was limited to articles written in English. Additionally, literature results that tested the effect of tobacco smoke on animals were included in this document for mechanisms of action to be explained. Represented in Table 1 is a summary of the number of scientific articles published on these topics as distributed over a period of 5 years as retrieved from PubMed. It is worth noting that cigarette and tobacco smoke are used interchangeably in this manuscript.

**Table 1:** Summary of publication distribution related to tobacco smoking

	**PubMed articles**								
	**Publications from 1807 to 2021**			**Publications during the last 5 years**					
**Keywords**	**Review articles**	**Systematic reviews**	**All publications**	**2016**	**2017**	**2018**	**2019**	**2020**	**5 years total**
“Tobacco”	12,810	1672	140,918	7587	7372	8119	8380	9038	40,496
“Tobacco” AND “male infertility”	40	7	115	3	7	12	9	8	39
“Tobacco” AND “sperm”	39	8	255	11	10	17	16	19	73
“Tobacco” AND “sperm DNA fragmentation”	2	0	16	1	2	2	0	1	6
“Tobacco” AND “sperm” AND “mutagenic”	3	0	9	0	0	0	0	0	0
“Tobacco” AND “sperm” AND “mutagen, mutagenic”	4	0	15	1	1	1	0	0	3
“Tobacco” AND “sperm, aneugen”	43	8	285	11	12	19	16	21	79

### Types of tobacco use and sources of tobacco smoking

Tobacco can be consumed in a wide variety of smoked and smokeless products. Tobacco smoking is the combustion of tobacco leaves and inhalation of the smoke. The most common methods of smoking tobacco include manufactured and hand-rolled cigarettes and cigars (worldwide, Europe, and Asia), waterpipes (Middle East, North Africa, and parts of Asia), pipes (worldwide), and more recently electronic nicotine delivery systems (North America and Europe) (Asma et al. 2015). Smokeless tobacco is consumed through the mouth or nose, without combustion or burning. The most common consumption of smokeless tobacco includes chewing tobacco (Africa, Asia, and North America), and both moist snuff (Asia, Middle East, North America, and South Africa) and dry snuff (Brazil, Europe, South and Central Asia, Nigeria, South Africa, and North America) (Asma et al. 2015). Primary exposure refers to inhalation of tobacco smoke or consumption of smokeless tobacco products (e.g., chewing tobacco, snuff) by the individual user. Secondary tobacco smoke exposure (also called environmental tobacco smoke) is inhalation of someone else’s, e.g., cigarette, cigar, or pipe smoke generated from combustion of tobacco products.

### Content of tobacco smoke

Firstly, tobacco use is of interest to male reproductive researchers as it contains over 7000 compounds, of which approximately 70 have been identified as carcinogenic (Hecht 2003; IARC 2012). Different toxicants present in tobacco smoke is reviewed in detail by Shihadeh et al. (Shihadeh et al. 2015; Nardone et al. 2019). Combustible tobacco products cause the emission, and hence exposure to gases, vaporized liquids, and particles through the processes of hydrogenation, pyrolysis, oxidation, decarboxylation, and dehydration (du Plessis et al. 2014). There are two phases of cigarette smoke with nicotine and tar released in the particulate phase, and carbon monoxide emitted in the gaseous phase (Hammond et al. 2006). A wide range of carcinogens and mutagens, including radioactive polonium, benzopyrene, dimethylbenzanthracene, naphthalene methylnaphthalene, polycystic aromatic hydrocarbons, and heavy metals such as cadmium, have been found in cigarette smoke (Hosseinzadeh Colagar et al. 2007; Richthoff et al. 2008; Dai et al. 2015). Some of these chemicals have been proven to be extremely detrimental to male fertility.

### Prevalence of male infertility

Infertility is defined as the inability of a couple to conceive after 1 year of unprotected intercourse (Lyu et al. 2017). Globally, ~15% of couples of reproductive age are affected by infertility and the distribution of infertility due to male factor ranges from 20 to 70% with the prevalence of male infertility in North America, Australia, and Central and Eastern Europe varied from 4.5 to 6%, 9%, and 8 to 12%, respectively (Agarwal et al. 2015). Data from the past 80 years suggests a temporal decline in semen quality. An earlier review (*N* = 14,947; 61 papers 1938–1991) reported that mean seminal volume significantly decreased from 3.40 to 2.75 ml between 1940 and 1990 with an estimated decline of −0.0130 ml/year (Carlsen et al. 1992). Similarly, a significant decrease in mean sperm concentration from 113 × 10^6^ to 66 × 10^6^/ml was observed between 1940 and 1990 with an estimated decrease of −0.934 × 10^6^/ml per year (Carlsen et al. 1992). Swan and colleagues (2000) expanded the work of Carlsen et al. (1992) by including an additional 47 studies and re-analyzed the data from 1934 to 1996 and reported significant declines in sperm concentration in the USA (approximately 1.5%/year) and Europe/Australia (approximately 3%/year) that were markedly greater than the average decline reported by Carlsen et al. (1992) (approximately 1%/year). Although there were limited data available, the review did not find a decline in sperm concentration in non-Western countries. The etiology remains unknown (termed idiopathic infertility) in approximately half of infertile males with the clinical sperm profile showing oligospermia, asthenospermia, teratozoospermia, or other sperm abnormalities (Lyu et al. 2017). A recent comprehensive meta-analysis reported that sperm counts, whether measured as sperm count or total sperm count, declined significantly among men from North America, Europe, and Australia during 1973–2011, with a 50–60% decline among men unselected by fertility, with no evidence of a “leveling off” in recent years. These findings strongly suggest a significant decline in male reproductive health, which has serious implications beyond fertility concerns (Levine et al. 2017).

### Epidemiology of tobacco use and male infertility

Numerous risk factors have been proposed for male infertility including erectile dysfunction, varicocele, congenital dysplasia, endocrine disorders, immune factors, sexually transmitted infections, and exposure to chemicals and radiation (Krausz, 2011). There is an emerging body of evidence to suggest the possible role of tobacco use in the etiology of male infertility due to changes in sperm parameters. A cross-sectional study of 1165 males aged 16–29 years from Estonia (*N* = 573), Latvia (*N* = 278), and Lithuania (*N* = 314) was conducted in 2003–2004 to investigate the sperm quality parameters in Baltic men (Erenpreiss et al. 2017). The median sperm concentration was 63 × 10^6^/ml and low semen quality was detected in 11–15% of the men. Smoking had an adverse impact on both sperm concentration and total sperm counts with smokers having significantly lower sperm concentrations (median 60 versus 66 × 10^6^/ml, *P* = 0.02) and lower total sperm counts, (177 versus 226 × 10^6^, *P* < 0.001) than non-smokers (Erenpreiss et al. 2017). Cross-sectional data cannot infer the direction of the relationship; therefore, data from robust longitudinal cohort designs provide stronger evidence on the temporality and possible causal association between tobacco use and male infertility. The Nanjing Medical University Longitudinal Investigation of Fertility and the Environment (NMU-LIFE) study investigated the effects of cigarette smoking on sperm quality among 1631 fertile men in China. The study reported a significant decrease in semen volume and total sperm count, and a significant increase in total motility and progressive motility in ever smokers of pack-years ≥10 compared with never smokers (Tang et al. 2019). Interestingly, the study reported an inverse dose-dependent relation between smoking pack-years and semen volume and total sperm count, and a positive dose-dependent relation between smoking pack-years and both total motility and progressive motility (Tang et al. 2019). The observed inverse dose-response association between cigarette smoking and sperm quality suggests a causal association. Moreover, the negative impact seems to be reversible as the detrimental effects of smoking on semen quality were not observed in men classified as former smokers (i.e., quit smoking for at least 6 months and had smoked for at least 1 year) (Tang et al. 2019). A recent systematic review and meta-analysis of 16 studies (*N* = 10,823 infertile male participants; 5257 smokers and 5566 non-smokers) showed that smokers were 26% more likely to have oligozoospermia than non-smokers (relative risk: 1.29; 95% CI: 1.05–1.59; *P* = 0.02) (Bundhun et al. 2019). Compared to non-smokers, morphological defects of spermatozoa (mean difference (MD): 2.44; 95% CI: 0.99–3.89; *P* = 0.001) was also significantly higher in smokers with significant head (MD: 1.76; 95% CI: 0.32–3.20; *P* = 0.02), neck (MD: 1.97; 95% CI: 0.75–3.18; *P* = 0.002), and tail (MD: 1.29; 95% CI: 0.35–2.22; *P* = 0.007) defects (Bundhun et al. 2019). Smoking did not affect the pH (MD: 0.04; 95% CI: −0.03–0.11; *P* = 0.30) or motility (RR: 1.42; 95% CI: 0.97–2.09; *P* = 0.07) of spermatozoa, or cause any disbalance in reproductive hormones (Bundhun et al. 2019). Another recent review presented data on more than 60 studies reporting the relationship between tobacco use and impaired seminal parameters in both animal and human models (Beal et al. 2017) Moreover, the available evidence in the review provides support for the notion that nicotine and chemical contained in tobacco can permeate the blood-testis barrier and act as a mutagen on human germ cells (Beal et al. 2017).

### Smoking and sperm function

**Fig. 1:**
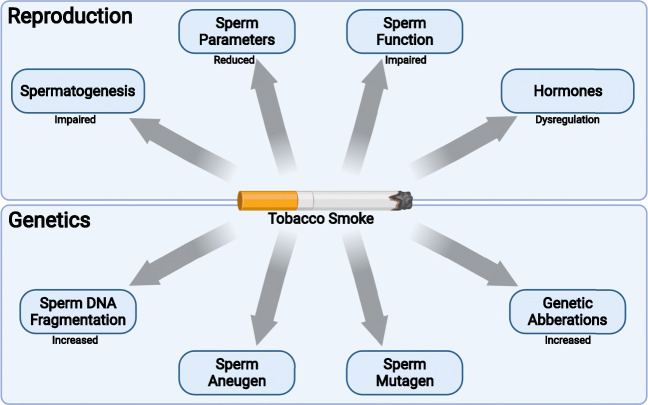
Effects of tobacco smoke on male fertility and its impact on genome integrity. Tobacco smoke has been shown to impair reproductive processes (spermatogenesis), semen or sperm parameters (concentration, semen volume), and sperm function (motility). In addition to tobacco smoke being regarded as an endocrine disruptor, it has been shown to cause increased sperm DNA fragmentation, sperm mutagenesis, and sperm aneuploidy, and can also lead to several genetic mutations, which cumulatively affects male fertility

Represented in Fig. 1 are some of the effects of tobacco smoke on male reproductive parameters and its impact on genome integrity. A systematic review and meta-analysis conducted by Bundhun et al. (Bundhun et al. 2019), which analyzed 10,823 infertile males (smokers and non-smokers) with a mean age of 26.5–40.5 years, found smoking to affect both sperm quality and sperm quantity. Smoking was associated with reduced sperm counts and increased morphologically abnormal sperm (head, neck, and tail defects). Motility, however, was a contentious outcome. This meta-analysis found tobacco smoking to have no effect on motility, whereas several other articles observed a negative effect (Lingappa et al. 2015; Asare-Anane et al. 2016; Sharma et al. 2016). Furthermore, in general, tobacco smoking has been found to reduce semen volume, sperm concentration, and total sperm count (Asare-Anane et al. 2016), although few studies have shown no effect (Trummer et al. 2002). Numerous mechanisms have been postulated regarding how these changes can come about. These are discussed in detail in later paragraphs. The consensus among authors is that cigarette smoking reduces all conventional (basic) sperm parameters, despite parameters such as motility. However, in many cases, progressive motility is reported, and it is found to be reduced (Mostafa et al. 2018; Boeri et al. 2019). As cigarette smoke has numerous compounds and carcinogens, studies have also been performed on the individual constituents to observe their effects on reproduction. Benzo[a]pyrene, a polycyclic aromatic hydrocarbon and powerful carcinogen and mutagen (Kaiserman and Rickert 1992), has been shown in vitro to induce higher percentages of acrosome reaction and hyperactivation with increasing concentrations of benzo[a]pyrene (Mukhopadhyay et al. 2010). Nicotine can cross the blood-testis barrier and is found in high concentrations within the seminal plasma of tobacco smokers (Pacifici et al. 1993; Abu-awwad et al. 2016). Nicotine has been shown by both in vitro and in vivo animal models to affect sperm parameters (Gandini et al. 1997; Cope and Gandini 1998; Oyeyipo et al. 2010, 2014; Ezzatabadipour et al. 2012). Oyeyipo et al. (2014) observed high levels of nicotine to induce premature acrosome reactions in spermatozoa and to reduce sperm motility and viability in vitro. Furthermore, cotinine, the main metabolite of nicotine, reduces sperm concentrations, total sperm count, and sperm motility (Vine et al. 1996). While almost all the human studies pertaining to the effects of smoking and sperm function has primarily considered cigarette smoking, other combustible tobacco products likely have the same impact such as hookah (or waterpipe) and cigars. Moreover, electronic cigarettes, although they are not classified as combustible products, can deliver high levels of nicotine similar to that of cigarettes (Farsalinos et al., 2014). There is no reason to expect these products to not impact sperm function and male infertility; however, empirical research is needed to evaluate its level of harm in comparison to cigarette smoking (Corona et al. 2020).

### Smoking and sperm production

Tobacco smoking reduces sperm concentration. This has been postulated to occur via several mechanisms including a reduction of spermatogenesis via impaired hormone and enzyme production, as well as histological damage to reproductive organs. Tobacco smoke has been proposed to be an endocrine disruptor. Blanco-Muñoz et al. (2012) were able to show that smokers have higher luteinizing hormone (LH), prolactin, and testosterone levels, but no effect on follicle-stimulating hormone (FSH), estradiol, and inhibin B. Other authors have also reported higher testosterone concentrations in smokers versus non-smokers, even after adjusting for age and anthropometric parameters (Field et al. 1994; Bønaa et al. 2003; Svartberg and Jorde 2007). In addition, clinical studies have shown a positive correlation between increased serum nicotine levels and higher levels of prolactin (Mendelson et al. 2003; Xue et al. 2010). Other studies, however, have not been able to show changes in hormone concentrations (Bundhun et al. 2019). Exposure to cigarette smoke has been observed to reduce the activity of sorbitol dehydrogenase and increase lactate dehydrogenase, both key enzymes in spermatogenesis and sperm maturation (Abdul-Ghani et al. 2014). In cigarette smoke–exposed young mice, histological assessment of the testis revealed smaller seminiferous tubules, atrophy, and tubular degeneration, as well as a reduction in the spermatogenic cell layer (La Maestra et al. 2015). These changes can negatively affect sperm production.

### Smoking and sperm DNA fragmentation

Tobacco smoking has been investigated by numerous studies to assess its effects on sperm DNA fragmentation. In a prospective randomized study analyzing 108 couples with unexplained infertility undergoing fertility treatment, 26 of the male counterparts were found to be smokers (Aboulmaouahib et al. 2018). Sperm DNA fragmentation and chromatin decondensation were analyzed via TUNEL (terminal deoxynucleotidyl transferase dUTP nick end labeling) assay and aniline blue staining respectively; both DNA fragmentation (26%) and chromatin decondensation (25%) were shown to be significantly higher in the smokers compared to the non-smokers (controls). Using the comet assay, La Maestro et al. (2015) observed both single- and double-stranded breaks to be significantly higher in mice exposed to cigarette smoke from birth to early childhood compared to controls.

### Mechanisms through which tobacco smoke affects male fertility

Most studies have reported several reproductive abnormalities such as impaired spermatogenesis, reduced semen quality, and altered sperm function in men that smoke tobacco (Martini et al. 2004; La Maestra et al. 2015). These outcomes are in part underlined by the increased formation of reactive oxygen species (ROS) leading to the development of oxidative stress (OS), DNA damage, and germ cell apoptosis. Although ROS is required for physiological processes (Haque et al. 2014), the abnormal accumulation thereof can lead to DNA strand breaks, peroxidation of unsaturated lipids, disruption of mitochondrial function, and oxidative damage to DNA (Fullston et al. 2017; Roychoudhury et al. 2017). Spermatozoa are vulnerable to ROS due to the presence of limited cytoplasmic antioxidants and limited repair mechanisms (Attia et al. 2014). Due to the translational and transcriptional inert nature of DNA, the DNA damage borne in sperm DNA persists (Attia et al. 2014).

Kumar et al. (2015) reported an increase in seminal ROS with a subsequent rise in sperm DNA fragmentation index and elevated 8-hydroxy-2-deoxyguanosine (8-OHdG) levels in the semen samples collected from smoking men. Another study reported a decrease in the activity of sperm glutathione peroxidase (GPx-1, 4) and a reduction in the mRNA expression of glutathione reductase in the spermatozoa of smoking men (Viloria et al. 2010). Several other authors have reported elevated levels of malondialdehyde and protein carbonyls in the semen of smokers and a reduction in the levels of glutathione-S-transferase and glutathione (Haque et al. 2014; Dai et al. 2015).

Many studies have reported that the contents of tobacco smoke, such as nicotine, can penetrate the blood-testis barrier and subsequently affect the process of spermatogenesis, either by affecting the genetic integrity or by altering hormone production (Toppari et al. 1996; Kumar et al. 2015; Aprioku and Ugwu 2016). Hence, environmental factors such as exposure to endocrine disruptors may affect the integrity of the sperm genome, especially the sperm nucleus where the DNA is loosely bound to histones in the nucleohistone compartment (Jeng 2014). Nicotine acts as an oxidizing agent that subsequently affects the sperm plasma membrane.

**Fig. 2:**
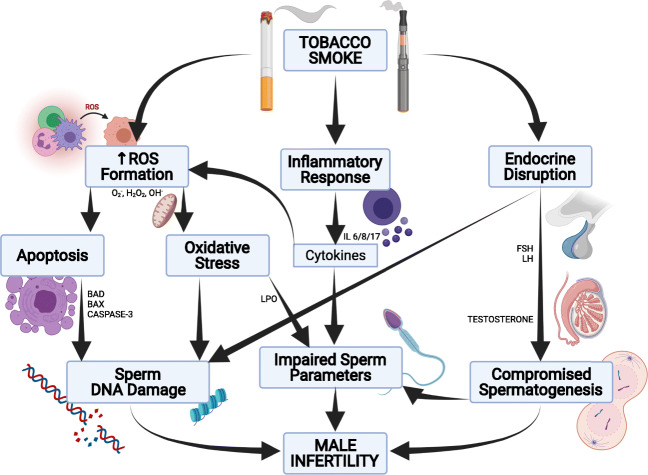
Mechanisms through which tobacco smoke affects male fertility. Tobacco smoke is known to cause increases in reactive oxygen species (ROS) formation and subsequently result in the development of oxidative stress. The excessive accumulation of ROS causes lipid peroxidation and increased DNA strand breaks, thereby resulting in altered sperm parameters/functions. Increased ROS formation have been shown to result in the development of apoptosis, where the levels of pro-apoptotic cytokines are increased and can further lead to elevated sperm DNA damage and ultimately impaired male fertility. Tobacco smoke substrates can disrupt follicle-stimulating hormone (FSH) and luteinizing hormone (LH) secretion from the anterior pituitary which can impact testosterone synthesis and spermatogenesis. Some of these toxicants can furthermore pass through the blood-testes-barrier, thereby directly affecting testosterone production/secretion and spermatogenesis. Hence, tobacco smoke is a known endocrine disruptor. Additionally, tobacco smoke has been shown to decrease the levels of nuclear protamine/protamination. Also, increased secretion of inflammatory cytokines has been shown in the sera of smokers, which in turn elevate the formation of ROS and consequently result in impaired sperm parameters and ultimately male infertility

The sperm plasma membrane is rich in polyunsaturated fatty acids (PUFA) which are highly susceptible to ROS; its invasion thereof leads to lipid peroxidation (Haque et al. 2014; Harlev et al. 2015). Lipid peroxidation occurs in 3 stages: initiation, propagation, and termination. During initiation, free radicals react with fatty acid chains to form the lipid peroxyl radical. Peroxyl radicals in turn react with fatty acids to produce free radicals and the reaction is thus propagated. In termination, the two radicals react with each other which lead to lipid break down (Omolaoye and Du Plessis 2018). Hence, an increase in an oxidizing agent consequently results in (i) sperm plasma membrane PUFA breakdown, (ii) development of oxidative stress, (iii) sperm DNA damage, (iv) impaired spermatogenesis, (v) reduced sperm production, (vi) impaired chromatin remodeling, and (vii) a decrease in the levels of nuclear protamination (Aitken et al. 2014). Looking at the effect of smoking on sperm DNA, it can be suggested that tobacco smoke is not only harmful to the active user, but also can affect the paternal genome to accumulate damage even before fertilization (Kumar et al. 2015). A summary of the mechanism through which tobacco smoke affects male fertility is represented in Fig. 2.

### Tobacco smoke as a sperm mutagen

Mutagenesis is a process by which the genetic information of an organism is changed by the production of a mutation. Mutation is the change in the structure of a gene as a result of exposure to mutagens. A mutagen, on the other hand, is any physical or chemical compound that can cause mutation. Although paternal age has always been emphasized as a major or significant contributor to increased male-related infertility and male-mediated inherited gene mutations (Sun et al. 2012; Kong et al. 2013), in recent years, however, the effect of paternal lifestyle such as tobacco smoking has been shown to contribute to germline mutations, which may not only have an adverse effect on the individual, but also on the offspring (Linschooten et al. 2013). Tobacco smoke has been shown to cause diverse reproductive abnormalities such as abnormal sperm parameters (as described in the section above), sperm DNA breaks, and chromosomal abnormalities, resulting in diverse consequences on the reproductive health of the smoker.

Both human and animal studies have identified tobacco smoke as a sperm mutagen (Yauk et al. 2007; Marchetti et al. 2011; Linschooten et al. 2013). Linschooten et al. in their study identified germline mutations in blood samples of complete mother-father-child traits. It was reported that the presence of these mutations were due to lifestyle. As paternally derived gene mutations increased dose dependently, that is, the offspring of fathers who smoked 6 months before pregnancy had a higher number of mutations compared to non-smoking fathers (Linschooten et al. 2013). A study that investigated the effect of main stream tobacco smoke on heritable genetic mutations in mice showed that animals exposed to mainstream tobacco smoke for a duration of 6 or 12 weeks exhibited a significant increase in germline mutation in spermatogonial stem cells, and the frequency of mutation was higher in the animals exposed for 12 weeks (Yauk et al. 2007). This suggests that tobacco smoke does not only affect the smoker himself, but the effect can extend to the non-smoking descendant. Marchetti et al. (2011) reported that the exposure of mice to short-term main stream tobacco smoke (2 weeks), and side stream tobacco smoke (second-hand smoke), both induced mutations at an expanded simple tandem repeats locus of mice spermatozoa. This further shows that tobacco smoke is a mutagen of spermatozoa, which could go beyond the smoker.

Findings have also indicated that tobacco smoke alters the microRNA expression in sperm and DNA methylation pattern in other cell types, some of which persisted even after cessation of smoking (Guida et al. 2015). Spermatozoa consist of a complex population of RNA including messenger RNA (mRNA) and micro RNA (miRNA), whose interaction can be used to study the mechanism by which mutagens affects spermatogenesis. A study reported a differential gene expression in the mRNA and miRNA of spermatozoa obtained from smoking men (Metzler-Guillemain et al. 2015). This is supported by another study that reported that tobacco smoke induces specific differences in the spermatozoa miRNA content of smoking men, and that the miRNA appear to be involved in the regulatory pathway that is important for healthy sperm and normal embryo development (Marczylo et al. 2012). Chen et al. also showed that there was alteration in sperm protein expression of mice exposed to tobacco smoke (6 weeks). The proteins affected were related to energy metabolism, reproduction, and structural development molecules (Chen et al. 2015). This shows that smoking can change the presentation and expression of genes that are required for spermatogenesis, which may consequently affect male fertility. Collectively, it can be acknowledged that the recommendation to cease tobacco smoking would not only be advantageous to the immediate primary smoker but would also prevent the development of genetic aberrations in offspring.

### Tobacco smoke as sperm aneugen

An aneugen is any substance that is capable of causing abnormality in the number of chromosomes. Aneugens affect cell division by interacting with the spindle apparatus rather than affecting or interacting with DNA (Faqi et al. 2017). The process of the occurrence of abnormal number of chromosome is called aneuploidy and it has been shown to occur in about 0.3% of live births and in 5–25% of mammalian zygotes (Hassold and Hunt 2001). Studies have highlighted that male partners contribute to about 50% of sex chromosome aneuploidy and that several substances can act as aneugens affecting the spermatogonia (Hassold and Hunt 2001; Robbins et al. 2005). Aneuploidies disrupt gene expression and balance; hence, most aneuploidies result in spontaneous abortion or stillbirth (Hassold and Hunt 2001), but those of the sex chromosomes and some autosomes are compatible to life, and they are associated with developmental disorders. Studies have shown that tobacco smoke can induce aneuploidy in male germ cells (Pereira et al. 2014). A study reported that the spermatozoa of smokers are likely to have an extra chromosome 3 (Pereira et al. 2014), and another study showed that tobacco smoke is associated with an increase in chromosome 1 and chromosome 7 disomies (Härkönen et al. 1999), while several other authors showed that tobacco smoke increases the occurrence of aneuploidies in sperm (Robbins et al. 1997; Shi et al. 2001; Faure et al. 2007). Additionally, the spermatozoa of smokers are likely to have multiple sex chromosomes, such as X-X, Y-Y, and X-Y disomies (Robbins et al. 1997). Hassold et al. suggested that smoking men have about two-fold increase in aneuploid sperm and that 0.15% of live births have autosomal trisomy. They further indicated that male smoking partners contribute to 5–10% of live birth autosomal trisomies (Hassold et al. 1996). Data have also suggested that approximately 0.02% of children born to male smokers would carry an abnormal number of autosomal chromosomes (Beal et al. 2017).

### Epidemiology of smoking and genetic aberrations

Earlier sections of this review presented recent evidence showing a strong association between tobacco use and decreased male fertility via a reduction in semen quality; however, the biological mechanism(s) by which tobacco use affects semen quality is not fully understood. Tobacco smoke contains nicotine, carbon monoxide, and heavy metals which are inhaled and transported throughout the body and can pass the blood-testis barrier to reach the seminal plasma (Hamad et al. 2014; Hua et al. 2019). In view that there are continuous cell divisions in the sperm cell differentiation and maturation process (Shukla et al. 2012), it is not surprising that the substances inhaled in tobacco smoke that reach the testes might be implicated in the pathogenesis of reduced sperm quality and male infertility. One proposed mechanism of action is that male infertility, as a result of abnormal spermatozoa, is caused by smoking-induced DNA damage due to activation of the checkpoint kinase 1 (Chk1) which facilitates S and G2 checkpoint arrest (Cui et al. 2016). A recent study in China, on a cohort of 841 smoking men and 287 non-smoking men, showed that abnormal head rates in the heavy smoking group and long-term smoking group were significantly increased compared with the non-smoking group (Cui et al. 2016). Moreover, increased sperm DNA fragmentation rates and decreased Chk1 expression were observed in the smoking group compared to the non-smoking group with an inverse association seen between sperm DNA fragmentation rates and the progressive motility and sperm concentration (Cui et al. 2016). Findings from this study suggest that cigarette smoking–induced decrements in semen quality might be caused by DNA damage and apoptosis leading to reduced Chk1 expression and sperm repair, and increased sperm apoptosis (Cui et al. 2016). Similarly, a recent study in Germany analyzed one hundred forty-one sperm samples (43 non-smokers and 98 heavy smokers) of couples undergoing intracytoplasmic sperm injection and reported significantly lower semen parameters and significantly higher protamine deficiency and sperm DNA fragmentation in heavy smokers (defined as at least one pack a day for 10 years or 2 packs a day for 5 years at least) compared to non-smokers (Amor et al. 2021). In addition, the studied sperm nuclear protein genes (i.e., H2B histone family member W, transition proteins 1 and 2, and protamine 1 and 2 genes) were differentially expressed and downregulated in the spermatozoa of heavy smokers compared to non-smokers (Amor et al. 2021). These findings suggest that tobacco smoking might reduce normal sperm function by altering the mRNA expression levels of H2BFWT, TNP1, TNP2, PRM1, and PRM2 genes. The available evidence suggests that tobacco smoke may be a human germ cell mutagen that reduces sperm quality through differential expression of important sperm nuclear protein genes.

### Innovative ways to curtail smoking

**Fig. 3:**
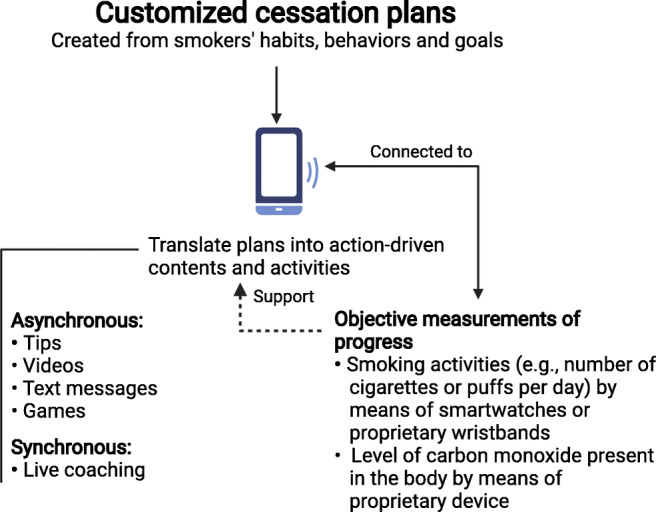
Innovative ways to curtail smoking

Tobacco smoke has a wide range of negative health consequences on human health including male fertility which can be reduced or even reversed with smoking cessation. Therefore, innovative approaches to smoking cessation are urgently needed at the population-level. In the last decade, an increasing number of digital solutions have been developed to curtail or help to stop smoking. These drug-free solutions offer new alternatives to the traditional nicotine patch (Fiore et al. 1994). The majority of these solutions leverage the large penetration of mobile devices that equip as many as 3.6 billion of people worldwide (Shoaib et al. 2018). Deployed as mobile applications, the solutions support smokers in the creation of customized plans according to the gender, age, smoking history, current consumption, and goals (Bricker et al. 2017; Chen et al. 2018). These plans are then enacted by different approaches and techniques as shown in Fig. 3. Some solutions are passive and include motivational short text messages, videos describing the benefits of a smoking-free life, or tips to help reduce cigarette consumption. Others involve the smoker in games or engage them in live coaching sessions (Ortis et al. 2020). In order to track cigarette consumption, mobile applications implement two types of loggers. The first type is subjective and requires the smoker to enter the smoking of each cigarette, while the second is objective and rely on smartwatches (Cole et al. 2017; Shoaib et al. 2018) or proprietary wristbands (Lopez-Meyer et al. 2013; Lu et al. 2019) to detect the number of cigarettes and puffs. The Pivot app goes one step further and employs a breath sensor connected to the smoker’s mobile device. This allows for objectively assessing the level of carbon monoxide, as marker of smoking, present in the body (Marler et al. 2020). Though some of these mobile applications and devices have been empirically evaluated, no systematic research has compared the efficacy of the different techniques.

### Strategies to manage male fertility in active smokers

The above section has highlighted some innovative ways through which active smokers can gradually curtail smoking and how the cessation thereof can help improve male fertility. The question remains, how about an active smoker who has refused to cease smoking? This section will briefly highlight some strategic ways to manage/improve male fertility in active tobacco smokers.

Some of the ways in which fertility can be improved in men who are not willing to quit smoking include (i) antioxidant therapy, (ii) nicotine replacement therapy, and (iii) assisted reproduction.

### Antioxidant therapy

Antioxidants are substances that inhibit oxidation of biologically relevant molecules, either by directly scavenging free radicals or by chelation of redox metals. They can be enzymatic (superoxide dismutase, catalase, and glutathione peroxidase), non-enzymatic (glutathione; n-acetylcysteine; vitamins E, A, and C; coenzyme Q10; carnitines; myo-inositol; lycopene), and antioxidants derived from nutrients (selenium, zinc, and copper), which can protect the body against OS (Zini et al. 2009; Walczak-Jedrzejowska et al. 2013; Barati et al. 2020). Studies, where antioxidant supplements were administered in idiopathic patients, reported an improvement in semen parameters (Ciftci et al. 2009; Ener et al. 2016; Smits et al. 2019). The supplements included vitamin E (Ener et al. 2016), selenium (Scott et al. 1998), N-acetylcysteine (Ciftci et al. 2009), and carnitine (Smits et al. 2019). In contrast to these findings, Steiner et al. (2020) reported that after administration of an antioxidant formulation containing 500 mg of vitamin C, 400 mg of vitamin E, 0.20 mg of selenium, 1000 mg of l-carnitine, 20 mg of zinc, 1000 μg of folic acid, and 10 mg of lycopene daily, to either treatment or placebo group, semen parameters or DNA integrity among men with male factor infertility did not improve (Steiner et al. 2020). The absence of effect may be due to the lack in large sample size or duration or due to other unknown causes. Despite the controversy, antioxidant therapy/supplements are currently being promoted to treat male factor infertility, as there are biological evidence that supports the hypothesis that antioxidants would improve male fertility.

### Nicotine replacement therapy

The aim of nicotine replacement therapy is to reduce motivation to consume tobacco and to also decrease the physiological and psychomotor withdrawal symptoms through the delivery of nicotine (Stead et al. 2008). This line of treatment has become well accepted as it is mostly recommended for people seeking pharmacological help to reduce or stop smoking (Le Foll et al. 2005). Although nicotine is the key active ingredient of tobacco smoke, studies have attributed the toxicity of tobacco smoking to tobacco’s other components, which are also responsible for widespread mortality and morbidity (Balfour 2004; Benowitz 2009). The most common and widely studied and implemented pharmacotherapy for managing nicotine dependence and withdrawal is the therapeutic use of nicotine-containing medications (Wadgave and Nagesh 2016). Nicotine replacement therapy products can be in the form of (i) gum, (ii) transdermal patch, (iii) nasal spray, (iv) oral inhaler, and (v) tablet.

The removal of all other tobacco components (released during combustion) will reduce the negative impact of tobacco smoke on male fertility, and the scrutinized monitoring of the nicotine level released through these nicotine replacement products will further lessen the adverse effect thereof.

### Assisted reproduction

Male factor infertility can be managed through Assisted Reproductive Technologies. Depending on the severity, specifically taking into account sperm concentration and motility, intrauterine insemination IUI, in vitro fertilization (IVF), or intracytoplasmic sperm injection (ICSI) can be recommended (Bhattacharya et al. 2001; Palermo et al. 2015).

## Conclusion and recommendation

Although the associated health risk of tobacco smoke is well documented, the global prevalence of smokers remains high. Tobacco smoke contains several toxic and mutagenic substances, some of which can cross the blood-testis barrier, and subsequently result in altered male reproductive parameters. Not only is the content of tobacco smoke implicated in the pathogenesis of male infertility; its contents have been shown to be a mutagen and an aneugen of spermatozoa, where the genetic mutations are not only seen in the spermatogonial stem cells of the smoker but also seen in the offspring. Findings have also shown that tobacco smoke can alter the microRNA expression in sperm, causing DNA methylation patterns in other cell types, some of which persisted even after cessation of smoking. This means that the adverse effect of tobacco smoking goes beyond the immediate smoker. Hence, it is recommended that male smokers should stop smoking tobacco well in advance of planning to have children and we have highlighted some novel smoking cessation solutions in the current review. Future studies should include data on smokeless tobacco and alternative ways in which nicotine is delivered into the system such as electronic cigarettes. The association between mutagenesis due to smoking and potential impacts on epigenetics should also be investigated further.
